# Hierarchical community detection via rank-2 symmetric nonnegative matrix factorization

**DOI:** 10.1186/s40649-017-0043-5

**Published:** 2017-09-08

**Authors:** Rundong Du, Da Kuang, Barry Drake, Haesun Park

**Affiliations:** 1 School of Mathematics, Georgia Institute of Technology, 686 Cherry Street, Atlanta, GA 30332-0160 USA; 2 Department of Mathematics, University of California, Los Angeles, 520 Portola Plaza, Los Angeles, CA 90095-1555 USA; 3 School of Computational Science and Engineering, Georgia Institute of Technology, 266 Ferst Drive, Atlanta, GA 30332-0765 USA; 4 Georgia Tech Research Institute, Georgia Institute of Technology, 250 14th Street, Atlanta, GA 30318 USA

**Keywords:** Community detection, Nonnegative matrix factorization, Constrained low rank approximation, Graph clustering

## Abstract

**Background:**

Community discovery is an important task for revealing structures in large networks. The massive size of contemporary social networks poses a tremendous challenge to the scalability of traditional graph clustering algorithms and the evaluation of discovered communities.

**Methods:**

We propose a divide-and-conquer strategy to discover hierarchical community structure, nonoverlapping within each level. Our algorithm is based on the highly efficient rank-2 symmetric nonnegative matrix factorization. We solve several implementation challenges to boost its efficiency on modern computer architectures, specifically for very sparse adjacency matrices that represent a wide range of social networks.

**Conclusions:**

Empirical results have shown that our algorithm has competitive overall efficiency and leading performance in minimizing the average normalized cut, and that the nonoverlapping communities found by our algorithm recover the ground-truth communities better than state-of-the-art algorithms for overlapping community detection. In addition, we present a new dataset of the DBLP computer science bibliography network with richer meta-data and verifiable ground-truth knowledge, which can foster future research in community finding and interpretation of communities in large networks.

## Background

Community detection is a key task in the study of large networks, which has recently become a very important area of research [1–3]. Although there are no generally accepted definitions of network communities, it is usually agreed that a community is defined as a group of nodes that have more intraconnections than interconnections. This high-level description defines community from a linkage point of view. In reality, a *functional* community in a network is usually based on some common affiliation, attribute, etc., which may not be directly related to linkage information. However, it has been shown that linkage-based communities usually have strong correlation with functional communities [4]. The purpose of community detection is to identify linkage-based communities or functional communities in a network.

Earlier work in this area mainly focused on finding linkage-based communities on small networks. Various measures have been defined and optimized for the difference between intraconnections and interconnections among network nodes, such as normalized cut, conductance, and others, using a variety of methods [4]. Manual examinations (such as visualization) of the results have typically been used in those studies, which may provide some valuable information and insights. Community detection on large networks is more challenging due to the following reasons: (1) many algorithms suitable for small networks are often not scalable; (2) there is a dearth of ground-truth communities defined for large networks and even for the datasets with ground truth, where the quality of the ground truth is often questionable; and (3) examining the results is nearly impossible. It has not been until recently [4] that ground-truth communities have been defined and studied in several real-world large-scale networks.

This paper introduces a scalable algorithm based on rank-2 symmetric nonnegative matrix factorization (rank-2 SymNMF) for large-scale hierarchical community detection. After summarizing some related works, we discuss the problem domain for our new results and our solutions for particular problems within that domain. Then, some highlights and speedup in our implementations are discussed, after which we present comprehensive experimental results to evaluate our new algorithm.

## Related work

The study of network community detection dates back to the well-known Kernighan–Lin algorithm from the early 1970s [5]. At that time, the network community detection problem was often formulated as a graph-partitioning problem, which aims at “dividing the vertices” into a predefined number of nonoverlapping “groups of predefined size, such that the number of edges lying between the groups is minimal” [6]. Many methods that produce good-quality solutions were proposed, but they were based on combinatorial optimization algorithms and were not scalable. Later, when it was discovered that graph partitioning is an important problem for balanced distribution of work loads in parallel computing, computer scientists developed many algorithms, such as METIS [7], SCOTCH [8], and Chaco [9], for graph partitioning of parallel communication problems. These algorithms usually follow a multilevel strategy, where a large graph is first coarsened to a smaller graph by recursively contracting multiple vertices into one vertex, and then the small graph is partitioned applying a method like the Kernighan–Lin algorithm, and finally the partition is mapped back to the original graph with some refinement. Most of these algorithms (e.g., all three we mentioned above) scale well to very large networks containing millions of nodes.

A spectral clustering method that minimizes normalized cut was proposed as an image-segmentation algorithm [10], and it soon became popular in the area of graph clustering. However, due to the time-consuming eigenvector/singular vector computation in this algorithm, it is not scalable to the case when the number of communities is large. The Graclus algorithm [11] by Dhillon, Guan, and Kulis solved this issue by utilizing the mathematical equivalence between general cut or association objectives (including normalized cut and ratio association) and the weighted kernel *k*-means objective [12] and applying a multilevel framework. Kuang, Ding, and Park discovered that the SymNMF (symmetric nonnegative matrix factorization) objective function is also equivalent to normalized cut and ratio association objective functions with a relaxation different from that in spectral clustering [13, 14]. This algorithm has better interpretability like many other NMF-based methods.

Girvan and Newman [15] produced pioneering work developing graph-partitioning/clustering methods from a community detection viewpoint, which finds “groups of vertices which probably share common properties and/or play similar roles within the graph” [6]. Around that time period, many new algorithms were invented. Later, Newman and Girvan [16] proposed the modularity measurement for community detection, on which the biggest family of community detection algorithms is based [17]. A scalable example in this family of algorithms is the Louvain algorithm [18]. Several algorithms such as Walktrap [19] and Infomap [20] are based on random walk on graphs, with the idea that in a random walk, the probability of staying inside a community is higher than going to another community. The paper [6] provides a comprehensive review of the algorithms that appeared up to 2010.

The early overlapping community detection algorithms [21, 22] were not effective on large graphs. Lancichinetti et al. [23] proposed a scalable overlapping community detection algorithm—*order statistics local optimization method* (OSLOM), which was based on a measurement similar to modularity but was able to handle overlapping communities. Yang and Leskovec studied the properties of large-scale overlapping communities [4] and proposed the BigClam algorithm [1]. They provided some large-scale datasets with ground-truth communities available to researchers, which have become standard test datasets. The BigClam algorithm seeks to fit a probabilistic generative model that satisfies certain community properties discovered in their studies [1, 24]. Whang et al. [2] proposed another overlapping community detection algorithm called NISE, based on seed set expansion, which starts with a seed set generated by Graclus or other methods and uses random walk to obtain overlapping communities. These algorithms that are dedicated to community detection have demonstrated better performance in terms of discovering ground-truth communities compared with the traditional graph-partitioning algorithms.

Recently, [17] proposed a new nonoverlapping community detection algorithm, *scalable community detection* (SCD). Network communities of good quality should have stronger intraconnections than interconnections. Previous algorithms measure such strength of connectivity only through the number of edges. The uniqueness of SCD is that it is based on a triangular structure of edges. The goal is to identify communities where each node forms more triangles with nodes inside the community than those formed with nodes outside the community.

On the other hand, nonnegative matrix factorization (NMF)-based methods exhibit superior interpretability in many application areas such as text mining [25, 26] and image analysis [27, 28]. In this paper, we will show that our NMF-based algorithm has competitive performance and scalability for community detection. Although our algorithm currently only handles nonoverlapping community detection, it has achieved comparable or even better-quality than the state-of-the-art overlapping community detection algorithms (such as BigClam) in our extensive tests. Our algorithm is inspired by SymNMF [13, 14] for graph clustering and HierNMF2 [25] for fast document clustering.

## Problem definition

As mentioned above, there is no universally accepted definition for network communities. Rather than defining community detection as optimizing some specific measurement criteria, we believe it would be more effective and flexible to understand the problem by clearly defining what makes a community detection result good.

In this paper, we focus on link-based community detection, and use functional communities (if known) as ground truth. In general, to evaluate link-based community detection results, we may ask several questions. The first is whether the result is coherent from the point of view of network links (Q1). There are many measurement scores defined based on the presumption that a community should have more intraconnections than interconnections. These measures include normalized cut, ratio cut, conductance, etc. The second question is whether the result agrees with prior knowledge, especially the ground truth if it is known (Q2). There have been largely two approaches. One is to manually analyze the results with some known meta-information (such as the entity each node represents), which is not scalable to large networks. Another approach is to compare the community detection result using some measures such as *F*1 score, which assumes the existence of ground truth. Finally, we would like to know whether the result reveals some new and useful information about the network (Q3). This is mostly relevant for the study of small networks [15]. For large networks, it is almost impossible to manually check all communities discovered. However, the answers to Q2 may guide our focus to more interesting parts of the network.

### Ground-truth communities

Ground-truth communities of large networks were not available to researchers until Yang and Leskovec [1] defined ground-truth communities (as found in SNAP^1^) for several real-world large networks, including several social networks, paper coauthorship networks, and product copurchase networks. In their work, the ground-truth communities are defined using functional communities already present in the data. For example, in social networks, user groups can be treated as communities; in paper coauthorship networks, two authors publishing in the same venue can be seen to be in the same community; in product copurchase networks, product category can be naturally used as communities. These functional communities are not necessarily directly related to network structures. For example, the product category is an inherent property of a product, which can never be affected by copurchasing activities. Therefore, it is not reasonable to expect that link-based community detection algorithms can fully recover functional communities. On the other hand, many studies show that there are close relations between link-based communities and functional communities. The paper [4] shows that many linkage-based measurements (such as normalized cut, conductance, etc.) also have good performance on functional communities. Also, the results of link-based community detection algorithms can sometimes recover functional communities.

Based on the above observations, we conclude that link-based community detection algorithms have the ability to partially recover functional communities, but such ability is inherently limited by the essential differences between functional communities and link-based communities. This should be kept in mind when comparing link-based community detection results against ground-truth communities.

### Overlapping vs nonoverlapping communities

Real-world network communities are usually overlapping. For example, it is common that one user joins a variety of groups in a social network. However, our current focus is on nonoverlapping community detection, since nonoverlapping community detection is also very useful for revealing the network structure, and our algorithm is designed to detect nonoverlapping communities efficiently. The results of a good-quality nonoverlapping community detection algorithm can be used as an effective starting point for overlapping community detection [2, 3].

## Hierarchical rank-2 symmetric NMF

We present an algorithm called HierSymNMF2 for hierarchical community detection. HierSymNMF2 uses a fast SymNMF algorithm [14] with rank 2 (SymNMF2) for binary community detection and recursively apply SymNMF2 to further binary split one of the communities into two communities in each step. This process is repeated until a preset number of communities is discovered, or there are no more communities that are worthy of any further binary split. Our approach starts with a low rank approximation (LRA) of the data based on the nonnegative matrix factorization (NMF), which reduces the dimension of the data while keeping key information. In addition, the results of NMF-based methods directly provide information regarding the assignment of data to clusters/communities.

Given the vast amounts of nonnegative data available for extracting critical information, the NMF has found a wealth of applications in such domains as image, text, and chemical data processing. It can be shown that applying algorithms to such data without constraining the solution can result in uninterpretable results such as negative chemical concentrations and possibly false negative and/or false positive detections, which could lead to meaningless results [29]. For text analytics, a corpus of text documents can be represented by a nonnegative term-document matrix. Likewise, for graph analytics, the nonnegative adjacency matrix is used as an input to NMF algorithms. NMF seeks an approximation of such nonnegative matrices with a product of two nonnegative low rank matrices. With various constraints and regularization terms on the NMF objective function, there are many variants of NMF, which are appropriate for a large variety of problem domains. A common formulation of NMF is the following:

1 $$\begin{aligned} \min _{W \ge 0,H\ge 0} \Vert X-WH\Vert _F \end{aligned}$$

where $X\in \mathbb {R}_+^{m\times n}$, $W\in \mathbb {R}_+^{m\times k}$, $H \in \mathbb {R}_+^{k\times n}$ ($\mathbb {R}_+$ is the set of all real nonnegative numbers), and $k\ll \min (m,n)$. In this formulation, each data item is represented by a column of the matrix *X*, and each column in the matrix *H* can be seen as a low rank representation of the data item. Nonnegativity constraints allow such a low rank representation to be more interpretable than other low rank approximations such as SVD. This formulation can be applied to areas such as document clustering [26] and can be solved efficiently for very large *m* and *n* [30]. However, when *k* reaches a value on the order of thousands, NMF algorithms become slow. To solve this issue, [25] developed a divide-and-conquer method that relies on rank-2 NMF, where $k=2$, which exhibits significant speedups. The framework of this divide-and-conquer method is shown in Algorithm 1. In this divide-and-conquer framework, the task of splitting one cluster into two clusters is performed by rank-2 NMF, which reduces the superlinear time complexity with respect to *k* to linear [25].

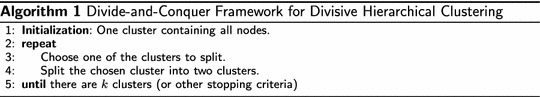



A variant of NMF, SymNMF [13, 14], which is the symmetric version of NMF, can be used for graph clustering. The formulation of SymNMF is

2 $$\begin{aligned} \min _{H\ge 0} \Vert S-HH^\text{T}\Vert _F \end{aligned}$$

where $S\in \mathbb {R}^{n\times n}$ is a symmetric similarity matrix of graph nodes: $H \in \mathbb {R}_+^{n\times k}$ and $k\ll n$. Some choices of the input matrix *S* for SymNMF are adjacency matrix $S^{\mathcal {G}}$ and normalized adjacency matrix $D^{-1/2}S^{\mathcal {G}}D^{-1/2}$, where $D = {\text {diag}}(d_1,\ldots ,d_n),$ and $d_i=\sum _{j=1}^n S^{\mathcal {G}}_{ij}$ is the degree of node *i*. When *S* is the adjacency matrix, (2) is a relaxation of maximizing the ratio association; when *S* is the normalized adjacency matrix, (2) is a relaxation of minimizing the normalized cut [13] (see Appendices A, B for a complete proof). SymNMF is an effective algorithm for graph clustering, but for large *k*, improvements in computational efficiency are necessary.

The algorithm we introduce in this paper uses the framework shown in Algorithm 1, where a cluster is a community, and the task of splitting a community is performed by our rank-2 version of SymNMF. The decision to choose the next node to split is based on a criterion discussed in the next section. In the following sections, we denote *S* as the similarity matrix representing a graph $\mathcal {G}$, and $S_c$ as the matrix representation of a community, i.e., a subgraph of $\mathcal {G}$ (the corresponding submatrix of *S*).

### Splitting a community using rank-2 SymNMF

Splitting a community is achieved by rank-2 SymNMF of $S_c\approx HH^\text{T}$ where $H\in \mathbb {R}_+^{n\times 2}$. The result *H* naturally induces a binary split of the community: suppose $H=(h_{ij})$, then

$$\begin{aligned} c_i= {\left\{ \begin{array}{ll} 1, & \quad h_{i1}>h_{i2};\\ 0, & \quad \text {otherwise}. \end{array}\right. } \end{aligned}$$

where $c_i$ is the community assignment of the *i*th graph node.

A formal formulation of rank-2 SymNMF is the following optimization problem:

3 $$\begin{aligned} \min _{H\ge 0} \Vert S-HH^\text{T}\Vert _F^2 \end{aligned}$$

where $H\in \mathbb {R}_+^{n\times 2}$. This is a special case of SymNMF when $k=2$, which can be solved by a general SymNMF algorithm [13, 14]. However, by combining the *alternating nonnegative least squares* (ANLS) algorithm for SymNMF from [14] and the fast algorithm for rank-2 NMF from [25], we can obtain a fast algorithm for rank-2 SymNMF.

First, we rewrite (3) into asymmetric form plus a penalty term [31] as follows:

4 $$\begin{aligned} \min _{W,H\ge 0} \Vert S-WH^\text{T}\Vert _F^2 + \alpha \Vert W-H\Vert _F^2 \end{aligned},$$

where *W* and $H\in \mathbb {R}_+^{n\times 2},$ and $\alpha >0$ is a scalar parameter for the tradeoff between the approximation error and the difference between *W* and *H*. Formulation (4) can be solved using a two-block coordinate descent framework, alternating between the optimizations for *W* and *H*. When we solve for *W*, (4) can be reformulated as

5 $$\begin{aligned} \min _{W\ge 0} \left\| \begin{bmatrix} H\\ \sqrt{\alpha } I_2 \end{bmatrix} W^\text{T} - \begin{bmatrix} S\\ \sqrt{\alpha }H^\text{T} \end{bmatrix} \right\| _F^2 \end{aligned}$$

where $I_2$ is the $2\times 2$ identity matrix. Similarly, when we solve for *H*, (4) can be reformulated as

6 $$\begin{aligned} \min _{H\ge 0} \left\| \begin{bmatrix} W\\ \sqrt{\alpha } I_2 \end{bmatrix} H^\text{T} - \begin{bmatrix} S\\\sqrt{\alpha }W^\text{T} \end{bmatrix} \right\| _F^2 \end{aligned}.$$

We note that both (5) and (6) are in the following form:

7 $$\begin{aligned} \min _{Y\ge 0} \Vert FY-G\Vert _F^2 \end{aligned},$$

where $F\in \mathbb {R}_+^{m\times 2}$, $G\in \mathbb {R}_+^{m\times n}$. This formulation can be efficiently solved by an improved active-set-type algorithm described in [25], which we call rank2nnls-fast. The idea behind rank2nnls-fast can be summarized as follows: the optimization problem (7) can be decomposed into *n* independent subproblems in the following form:

8 $$\begin{aligned} \min _{\varvec{y} \ge 0} \Vert F\varvec{y} -\varvec{g}\Vert _2^2 \end{aligned}$$

where $\varvec{y}$ and $\varvec{g}\in \mathbb {R}_+^2$, where $Y=[\varvec{y}_1,\ldots ,\varvec{y}_n]$, and $G=[\varvec{g}_1,\ldots ,\varvec{g}_n]$. To solve (8) efficiently, we note that when $\varvec{g}\ne 0$, there will be only three possible cases for $\varvec{y}=[y_1,y_2]$, where only one of $y_1$ and $y_2$ is 0 or both are positive. These three cases can easily be solved by the usual least-squares algorithms, e.g., normal equations. Details can be found in Algorithm 2 in [25].

### Choosing a node to split based on normalized cut

The “best” community to split further is chosen by computing and comparing *splitting scores* for all current communities corresponding to the leaf nodes in the hierarchy. The proposed splitting scores are based on normalized cut. We make this choice because (1) normalized cut determines whether a split is structurally effective since it measures the difference between intraconnections and interconnections among network nodes; and (2) for SymNMF, when *S* is the normalized adjacency matrix, the SymNMF objective function is equivalent to (a relaxation of) minimizing the normalized cut, which is the preferred choice in graph clustering [14].

Suppose we have a graph $\mathcal {G}=(V,E)$, where the weight of an edge (*u*, *v*) is *w*(*u*, *v*). Note that for an unweighted graph, $w(u,v)=1$ if edge $(u,v)\in E$; otherwise, $w(u,v)=0$. Let $A_1,\ldots ,A_k$ be *k* pairwise disjoint subsets of *V*, where $\bigcup _{i=1}^k A_i=V$; then, the normalized cut of the partition $(A_1,\ldots ,A_k)$ is defined as

9 $$\begin{aligned} {{\mathrm{ncut}}}(A_1,\ldots ,A_k) = \sum _{i=1}^k \frac{{{\mathrm{out}}}(A_i)}{{{\mathrm{within}}}(A_i)+{{\mathrm{out}}}(A_i)} \end{aligned}$$

where

10 $$\begin{aligned} {{\mathrm{within}}}(A_i)= \sum _{u,v\in A_i}w(u,v) \end{aligned}$$

which measures the number of edges inside the subgraph induced by $A_i$ (intraconnection); and

11 $$\begin{aligned} {{\mathrm{out}}}(A_i)= \sum _{u\in A_i,v\in V\setminus A_i}w(u,v) \end{aligned}$$

measures the number of edges between $A_i$ and the remaining nodes in the graph (interconnection). Note that in the definition of ${{\mathrm{within}}}(A_i)$ (10), each edge within $A_i$ is counted twice. In the special case, $k=2$, we have

12 $$\begin{aligned} {{\mathrm{out}}}(A_1)=\sum _{u\in A_1, v\in A_2}w(u,v)={{\mathrm{out}}}(A_2)\mathop {=}\limits ^{\mathrm{def}} \mathrm{cut}(A_1,A_2) \end{aligned}$$

From Eq. (9), it is evident that when each community has many more intraconnections than interconnections, there is a small normalized cut.

**Fig. 1:**
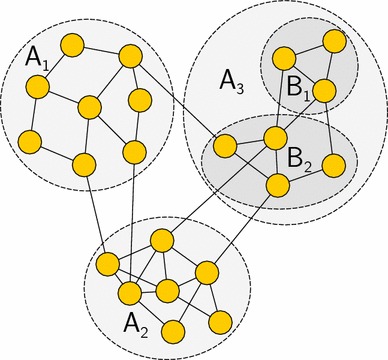
A graph for illustrating normalized cut and our splitting criteria. The structure of the graph is inspired by Figure 1 from [15]

For example, the graph shown in Fig. 1 originally has three communities $A_1$, $A_2$ and $A_3$, and the corresponding normalized cut is

$$\begin{aligned} {{\mathrm{ncut}}}(A_1,A_2,A_3) & = \frac{{{\mathrm{out}}}(A_1)}{{{\mathrm{within}}}(A_1)+{{\mathrm{out}}}(A_1)}+ \frac{{{\mathrm{out}}}(A_2)}{{{\mathrm{within}}}(A_2)+{{\mathrm{out}}}(A_2)} \\ & \quad + \frac{{{\mathrm{out}}}(A_3)}{{{\mathrm{within}}}(A_3)+{{\mathrm{out}}}(A_3)} \end{aligned}$$

The community $A_3$ is now split into two smaller communities $B_1$ and $B_2$ and normalized cut can be used to measure the goodness of this split. We consider three possibilities: (1) isolate $A_3$ and compute normalized cut of the split as

$$\begin{aligned} {{{\mathrm{ncut}}}}|_{A_3}(B_1,B_2)= \frac{{{{\mathrm{out}}}}|_{A_3}(B_1)}{{{\mathrm{within}}}(B_1)+{{{\mathrm{out}}}}|_{A_3}(B_1)}+ \frac{{{{\mathrm{out}}}}|_{A_3}(B_2)}{{{\mathrm{within}}}(B_2)+{{{\mathrm{out}}}}|_{A_3}(B_2)} \end{aligned}$$

where the subscript $A_3$ means only consider the edges inside $A_3$. We denote the above criterion by ncut_local. (2) A more global criterion is to also consider the edges that go across $A_3$:

$$\begin{aligned} {{\mathrm{ncut}}}(B_1,B_2)= \frac{{{\mathrm{out}}}(B_1)}{{{\mathrm{within}}}(B_1)+{{\mathrm{out}}}(B_1)}+ \frac{{{\mathrm{out}}}(B_2)}{{{\mathrm{within}}}(B_2)+{{\mathrm{out}}}(B_2)} \end{aligned}$$

This criterion is denoted by ncut_global. (3) Minimize the global normalized cut using a greedy strategy. Specifically, choose the split that results in the minimal increase in the global normalized cut:

$$\begin{aligned}&{{\mathrm{ncut}}}(A_1,A_2,B_1,B_2)- {{\mathrm{ncut}}}(A_1,A_2,A_3)\\ \quad \quad =\frac{{{\mathrm{out}}}(B_1)}{{{\mathrm{within}}}(B_1)+{{\mathrm{out}}}(B_1)}+ \frac{{{\mathrm{out}}}(B_2)}{{{\mathrm{within}}}(B_2)+{{\mathrm{out}}}(B_2)}- \frac{{{\mathrm{out}}}(A_3)}{{{\mathrm{within}}}(A_3)+{{\mathrm{out}}}(A_3)} \end{aligned}$$

We denote this criterion by ncut_global_diff and will compare the performance of these three criteria in later sections.

## Implementation

In the previous work on rank-2 NMF [32] that takes a term-document matrix as input in the context of text clustering, *sparse–dense matrix multiplication* (SpMM) was the main computational bottleneck for computing the solution. However, this is not the case with rank-2 SymNMF or HierSymNMF2 for community detection problems on typical large-scale networks. Suppose we have an $n \times n$ adjacency matrix with *z* nonzeros as an input to rank-2 SymNMF. In Algorithm 2, i.e., Nonnegative Least Squares (NLS) with two unknowns, SpMM costs 2*z* floating-point operations (flops), while searching for the optimal active set (abbreviated as opt-act) costs 12*n* flops. Of the 12*n* flops for opt-act, 8*n* flops are required for solving *n* linear systems each of size $2 \times 2$ corresponding to line 1 in Algorithm 2, and the remaining 4*n* flops are incurred by lines 4–5. Note that comparison operations and the memory I/O required by opt-act are ignored.

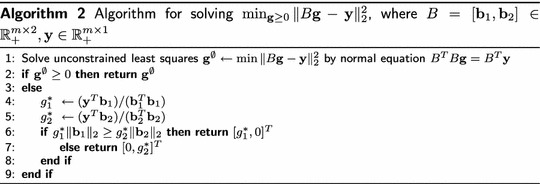



The above rough estimation of computational complexity reveals that if $z \le 6n$, or equivalently, if each row of the input adjacency matrix contains no more than 6 nonzeros on average, then SpMM will not be the major bottleneck of the rank-2 SymNMF algorithm. In other words, when the input adjacency matrix is extremely sparse, which is the typical case we have seen on various datasets (Table 1), then further acceleration of the algorithmic steps in opt-act will achieve higher efficiency.

**Fig. 2:**
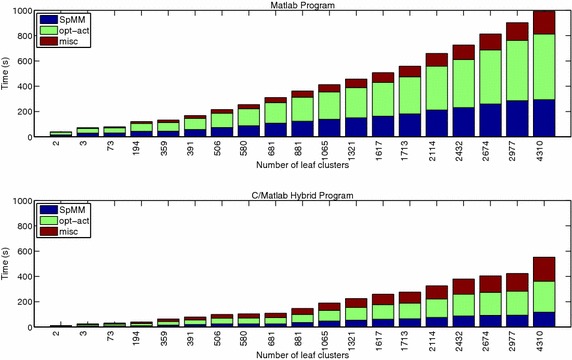
Runtime for SpMM, opt-act, and other algorithmic steps (indicated as “misc”) in the HierSymNMF2 algorithm. The experiments were performed on the *DBLP06* dataset. The plots show the runtime for generating various numbers of leaf nodes. *Upper* timing results for the Matlab program; *lower* timing results for the C/Matlab hybrid program

Figure 2 (upper) shows the proportions of runtime corresponding to SpMM, opt-act, and other algorithmic steps implemented in Matlab, which demonstrate that both SpMM and opt-act are the targets for performance optimization.

### Multithreaded SpMM

SpMM is a required routine in lines 1, 4, and 5 of Algorithm 2. The problem can be written as

13 $$\begin{aligned} Y \leftarrow A \cdot X, \end{aligned}$$

where $A \in \mathbb {R}^{n \times n}$ is a sparse matrix and $X,Y \in \mathbb {R}^{n \times k}$ are dense matrices.^2^

Most open-source and commercial software packages for sparse matrix manipulation have a single-threaded implementation for SpMM, for example, Matlab^3^, Eigen^4^, and Armadillo^5^ (the same is also true for SpMV, *sparse matrix–vector multiplication*). For the Intel Math Kernel Library^6^, while we are not able to view the source, our simple tests have shown that it can exploit only one CPU core for computing SpMM. Part of the reason for the lack of parallel implementation of SpMM in generic software packages is that the best implementation for computing SpMM for a particular matrix *A* depends on the sparsity pattern of *A*.

In this paper, we present a simple yet effective implementation to compute SpMM for a matrix *A* that represents an undirected network. We exploit two important facts in order to reach high performance:

- Since the nodes of the network are arranged in an arbitrary order, the matrix *A* is not assumed to have any special sparsity pattern. Thus, we can store the matrix *A* in the commonly used generic storage, the *compressed sparse column* (CSC) format, as is practiced in the built-in sparse matrix type in Matlab. As a result, nonzeros of *A* are stored column-by-column.
- The matrix *A* is symmetric. This property enables us to build an SpMM routine for $A^\text{T}X$ to compute *AX*.

The second fact above is particularly important: When *A* is stored in the CSC format, computing *AX* with multiple threads would incur atomic operations or mutex locks to avoid race conditions between different threads. Implementing multithreaded $A^\text{T}X$ is much easier, since $A^\text{T}$ can be viewed as a matrix with nonzeros stored row-by-row, and we can divide the rows of $A^\text{T}$ into several chunks and compute the product of each row chunk with *X* on one thread. Our customized SpMM implementation is described in Algorithm 3.

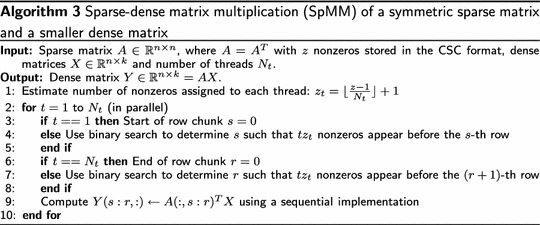



In addition, the original adjacency matrix often has the value “1” as every nonzero entry, that is, all the edges in the network carry the same weight. Thus, multiplication operations are no longer needed in SpMM with such a sparse matrix. Therefore, we have developed a specialized routine for the case where the original adjacency matrix is provided as input to HierSymNMF2.

### C/Matlab hybrid implementation of opt-act

The search for the optimal active set, opt-act, is the most time-consuming step in the algorithm for NLS with two columns (Fig. 2 (lower)) when the input matrix is extremely sparse. Our overall program was written in Matlab, and the performance of the opt-act portion was optimized with native C code. The optimization exploits multiple CPU cores using OpenMP, and the software vectorization is enabled by calling AVX (*advanced vector extensions*) intrinsics.

It turns out that a C/Matlab hybrid implementation is the preferred choice for achieving high performance with native C code. Intel CPUs are equipped with AVX vector registers, since the Sandy Bridge architecture and these vector registers are essential for applying the same instructions to multiple data entries (known as *instruction-level parallelism* or SIMD). For example, a 256-bit AVX register can process four double-precision floating point numbers (64-bit each) in one CPU cycle, which amounts to four times speed-up over a sequential program. AVX intrinsics are external libraries for exploiting AVX vector registers in native C code. These libraries are not part of the ANSI C standard but retain mostly the same interface on various operating systems (Windows, Linux, etc). However, to obtain the best performance from vector registers, functions in the AVX libraries often require the operands having aligned memory addresses (32-byte aligned for double precision numbers). The function calls for aligned memory allocation, which is completely platform dependent for native C code, means that our software would not be easily portable across various platforms if aligned memory allocation were managed in the C code. Therefore, in order to strike the right balance between computational efficiency and software portability, our strategy is to allocate memory within Matlab for the vectors involved in opt-act, since Matlab arrays are memory aligned in a cross-platform fashion.

Finally, note that the opt-act step in lines 1, 4, and 5 of Algorithm 2 contains several division operations, which cost more than 20 CPU cycles each and are much more expensive than multiplication operations (1 CPU cycle). This large discrepancy in time cost would be substantial for vector–scalar operations. Therefore, we replace vector–scalar division, in the form of $\mathbf {x}/{\alpha }$ where $\mathbf {x}$ is a vector and $\alpha$ is a scalar, by vector–scalar multiplication, in the form of $\mathbf {x} \cdot (1/{\alpha })$.

## Experiments

### Methods for comparison

We compare our algorithm with some recent algorithms mentioned in the “Related work” section. We use eight threads for all methods that support multithreading. For NISE, we are only able to use one thread because its parallel version exits with errors in our experiments. For all the algorithms, default parameters are used if not specified. To better communicate the results, below are the labels that denote each algorithm, which will be used in the following tables:

- h2-n(g)(d)-a(x): These labels represent several versions of our algorithm. Here h2 stands for HierSymNMF2, n for the ncut_local criterion, ng for the ncut_global criterion, and ngd for the ncut_global_diff criterion (see previous sections for the definitions of these criteria); ‘a’ means that we compute the real normalized cut using the original adjacency matrix; and ‘x’ indicates that an approximated normalized cut is computed using the normalized adjacency matrix, which usually results in faster computations. We stop our algorithm after $k-1$ binary splits where *k* is the number of communities to find. Theoretically, this will generate *k* communities. However, we remove fully disconnected communities, as outliers since they are often far from being significant because of their unusually small size and they correspond to all-zero submatrices in the graph adjacency matrix, which does not have a meaningful rank-2 representation. Therefore, the final number of communities are usually slightly smaller than *k*, as will be shown in “Experiment results” section.
- SCD: SCD algorithm [17].
- BigClam: BigClam algorithm [1].
- Graclus: Graclus algorithm [11].
- NISE: An improved version of NISE that is published in 2016 [3].

### Evaluation measures

#### Internal measures: average normalized cut/conductance

Normalized cut (9) is a measurement of the extent that communities have more intraconnections than interconnections and is shown to be an effective score [4]. Since our algorithm implicitly minimizes the normalized cut, it is natural to use normalized cut as an internal measure of community/clustering quality. One drawback of normalized cut is that it tends to increase when the number of communities increases. In Appendix B, we prove that the normalized cut strictly increases when one community is split into two. In practice, we observed that the normalized cut increases almost linearly with respect to the number of communities. Some community detection algorithms automatically determine the number of communities; hence, it is not fair to compare normalized cut for such algorithms against others that detect a preassigned number of communities. Therefore, it makes more sense to use the average normalized cut, i.e., the normalized cut divided by the number of communities. In addition, since the average normalized cut can be treated as a per-community property, it also applies to overlapping communities. Given *k* communities $A_1,\ldots ,A_k$ (which may be overlapping), we define the average normalized cut as

14 $$\begin{aligned} {{\mathrm{AvgNcut}}}(A_1,\ldots ,A_k) =\frac{1}{k} \sum _{i=1}^k \frac{{{\mathrm{out}}}(A_i)}{{{\mathrm{within}}}(A_i)+{{\mathrm{out}}}(A_i)} \end{aligned}$$

Conductance [33], which is shown to be an effective measure [4], is defined for a community as ${{\mathrm{Conductance}}}(A_i)=\frac{{{\mathrm{out}}}(A_i)}{{{\mathrm{within}}}(A_i)+{{\mathrm{out}}}(A_i)}$. Hence the average normalized cut is actually equal to the average conductance (per community).

#### External measures: precision, recall, and *F*-score

Alternatively, we can measure the qualities of detected communities by comparing them with ground truth. Suppose *k* communities $A_1,\ldots ,A_k$ were detected, and the ground truth has $k'$ communities $B_1,\ldots ,B_{k'}$. We compute the confusion matrix $C=(c_{ij})_{k\times k'}$, where $c_{ij}=|A_i\cap B_j|$. Then, pairwise scores can be defined as

$$\begin{aligned} {{\mathrm{Precision}}}(A_i,B_j) &={{\mathrm{recall}}}(B_j,A_i)=\frac{c_{ij}}{|A_i|} \\ {{\mathrm{Recall}}}(A_i,B_j) &={{\mathrm{precision}}}(B_j,A_i)=\frac{c_{ij}}{|B_j|}\\ F_1(A_i,B_j) & =F_1(B_j,A_i)=\frac{2c_{ij}}{|A_i|+|B_j|}\\ F_{\beta }(A_i,B_j) & = F_{1/\beta }(B_j,A_i)=\frac{(1+\beta ^2)c_{ij}}{|A_i|+\beta ^2|B_j|}. \end{aligned}$$

Although a global best match (i.e., finding a one-to-one mapping) between detected communities and ground-truth communities would be ideal, finding such a match is time consuming. We used per-community best match as a heuristic alternative. Specifically, we define the average $F_1$ score [1] as

$$\begin{aligned} F_1=\frac{1}{2}\left( \frac{1}{k}\sum _{i=1}^k\max _jF_1(A_i,B_j) + \frac{1}{k'}\sum _{j=1}^{k'}\max _iF_1(B_j,A_i) \right) . \end{aligned}$$

The average precision and average recall can be defined in a similar way. When comparing detected communities against ground truth, we remove nodes without ground-truth labels from the detected communities to achieve meaningful comparisons.

### Datasets

The data used for the experimental results of this paper are mostly from SNAP datasets [4, 34]. In our study, we found that the ground-truth information in SNAP is incomplete; for example, a large percentage of nodes do not belong to any ground-truth community. Table 1 shows some statistics regarding the number of communities to which each node belongs.

**Table 1:** Some statistics for ground-truth communities from SNAP

Dataset	#Nodes	#Edges	Nodes that belong to		Nodes that belong to		
			0 Community		1 Community		
			Count	%	Count	%	Rel %
*DBLP06*	317,080	1,049,866	56,082	17.69	150,192	47.37	57.55
*Youtube*	1,134,890	2,987,624	1,082,215	95.36	32,613	2.87	61.91
*Amazon*	334,863	925,872	14,915	4.45	10,604	3.17	3.31
*LiveJournal*	3,997,962	34,681,189	2,850,014	71.29	394,234	9.86	34.34
*Friendster*	65,608,366	1,806,067,135	57,663,417	87.89	3,546,017	5.40	44.63
*Orkut*	3,072,441	117,185,083	750,142	24.42	128,094	4.17	5.52

The last few columns show the number of nodes that do not belong to any communities and the number of nodes that belong to only one community. The “Rel %” is the number of nodes that belong to one community divided by the number of nodes that belong to at least one community

Although all of these datasets can be conveniently accessed on the SNAP website as a graph with ground-truth communities, *DBLP06* is the only dataset with a complete raw dataset openly available to the public. The other five datasets (*Youtube*, *Amazon*, *LiveJournal*, *Friendster*, and *Orkut*) were obtained by crawling the web, and they are far from being complete. Crawling large complex graphs is challenging by itself that may need extensive and specialized research efforts. We do not aim to solve this issue in this paper. The *Orkut* and *Youtube* datasets can be acquired from [35]. Detailed descriptions are available explaining the crawling procedure and analysis of the completeness. It has been concluded that the *Orkut* and *Youtube* datasets are not complete. Such incompleteness in crawled datasets is expected due to intrinsic restrictions of web crawling such as rate limit and privacy protection. The *Friendster* data were crawled by the ArchiveTeam, and the *LiveJournal* data come from [36]. The *Amazon* data were crawled by the SNAP group [37]. However, information on how the data were collected and processed and the information on analysis of data completeness are not available.

Possible reasons that many nodes in these datasets do not belong to any communities are (1) SNAP removed communities with less than three nodes, which caused some nodes to “lose” their memberships; (2) the well-known incompleteness of crawled datasets; (3) for social networks (*Youtube*, *LiveJournal*, *Friendster*, and *Orkut*), it is common that a user does not join any user groups; (4) SNAP used the dataset from [36] to generate the *DBLP06* dataset, which was published in 2006. At that time, the DBLP database was not as mature and complete as it is today. Another issue of the above datasets is that all nodes are anonymized, which ensures protection of user privacy, but limits our ability to interpret community detection results.

The DBLP data are openly accessible, and are provided using a highly structured format—XML. We reconstructed the coauthorship network and ground-truth communities from a recent DBLP snapshot to obtain a more recent and complete DBLP dataset with all of the meta-information preserved (see the following subsection). Although the other datasets which we currently cannot improve are also valuable, our goal is to obtain new information from comparison of community detection results and ground-truth communities, rather than simply recovering the ground-truth communities.

#### Constructing the DBLP15 dataset

**Fig. 3:**
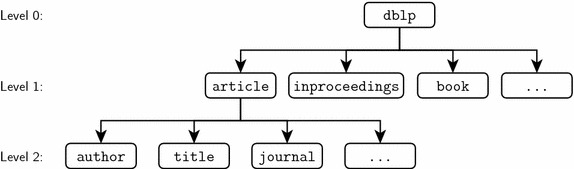
Structure of dblp.xml

DBLP is an online reference for bibliographic information on major computer science publications [38]. As of June 17, 2015, DBLP has indexed 4316 conferences, 1417 journals, and 1,573,969 authors [39]. The whole DBLP dataset is provided in a well-formatted XML file. The snapshot/release version of the data we use can be accessed at http://dblp.dagstuhl.de/xml/release/dblp-2015-06-02.xml.gz. The structure of this XML file is illustrated in Fig. 3. The root element is the dblp element. We call the children of the root elements *Level 1 elements* and the children of Level 1 elements *Level 2 elements*, and so on. Level 1 elements represent the individual data records [40], such as article and book, etc. Since publication–venue relation makes more sense for the journal and conference papers, and these two types of publications occupy most of DBLP, we consider only article and inproceedings elements when constructing our dataset. Level 2 elements contain the meta-information about the publications, such as title, authors, journal/proceeding names, etc.

Our goal is to obtain a coauthorship network and ground-truth information (venue–author relation) from the XML file. Although the XML file is highly structured, such a task is still not straightforward due to the ambiguity of entities, such as conflicts or changes of author names, various abbreviations, or even journal name change. DBLP resolves the author ambiguity issue by means of a unique number for each author. However, the venue ambiguity is still an issue in DBLP: there are no unique identifiers for venues. Fortunately, each record in DBLP has a unique key, and most paper keys contain the venue information as follows:

$$\underbrace {{{\texttt{journals}}}}_{\begin{subarray}{l} {\text{venue}} \\ {\text{type}} \end{subarray} }/\underbrace {{{\texttt{siamsc}}}}_{\begin{subarray}{l} {\text{venue}} \\ {\text{identifier}} \end{subarray} }/\overbrace {{{\texttt{KimP11}}}}^{\begin{subarray}{l} {\text{publication}} \\ {\text{identifier}} \end{subarray} }$$

However, there are still a few exceptions. To examine the validity of venue identifiers efficiently, we manually examine the identifiers not listed in the journal and conference index provided by the DBLP website, since such indices seem to be maintained by humans and assumed to be reliable. Using this process, we found 5240 unique venues (journals or conferences).

Now unique identifiers for both authors and venues make extracting the network and community information very reasonable. The next step is to create a node for each author, and create a link between two authors if they have ever coauthored in the same publication. For community information, each venue is a community, and an author belongs to a community if he/she has published in the corresponding venue.

A few authors do not have any coauthor in the DBLP database, and become isolated nodes in the generated network. Thus, we remove these authors. However, after removing those authors, some venues/communities become empty because all of their authors are removed. Hence, we remove those empty communities. After this cleaning, we obtained 1,509,944 authors in 5147 communities (venues).

This cleaned network has 51,328 (weakly) connected components, where the largest connected component contains 1,357,781 nodes, which makes 89.9% of all nodes. The remaining 51,327 connected components are all small, the largest of which has only 37 nodes. We take the largest connected component as the network to study. By extracting the largest connected component, we obtain a network with 1,357,781 nodes, 6,369,212 edges and 5146 ground-truth communities. The ground-truth communities were divided into connected components, obtaining 93,824 communities. The divided ground-truth communities were used for comparison with detected communities.

The new *DBLP15* dataset is available at https://github.com/smallk/smallk_data/tree/master/dblp_ground_truth.

### Experiment results

We run our experiments on a server with two Intel E5-2620 processors, each having six cores, and 377-GB memory. The results are listed in Tables 2, 3, 4, 5, 6, 7, 8, and 9.

**Table 2:** *DBLP06*: internal measures

Algorithm	Number of clusters	Coverage (%)	Algorithm time (s)	Total time (s)	Average Ncut
h2-n-a	4982	98.57	612.99	614.12	0.2089
h2-n-x	4981	98.55	587.98	589.10	0.2174
h2-ng-a	4984	98.48	921.99	923.14	0.1922
h2-ng-x	4982	98.50	872.48	873.64	0.1921
h2-ngd-a	4986	98.64	882.27	883.41	0.1767
h2-ngd-x	4984	98.66	908.31	909.46	0.1774
SCD	139,986	100.00	1.89	4.52	0.8091
BigClam	5000	90.57	N/A	230.59	0.6083
Graclus	5000	100.00	161.70	162.01	0.2228
NISE	5463	99.33	501.38	501.53	0.2026

**Table 3:** *DBLP06*: external measures

Algorithm	Number of clusters	*F*1	Precision	Recall	Reverse precision	Reverse recall
h2-n-a	3312	0.4355	0.8804	0.5242	0.9005	0.4030
h2-n-x	3298	0.4236	0.8855	0.5071	0.9007	0.3937
h2-ng-a	3211	0.4417	0.8708	0.5492	0.8490	0.3996
h2-ng-x	3118	0.4374	0.8742	0.5497	0.8574	0.3898
h2-ngd-a	3192	0.4577	0.8575	0.5800	0.8719	0.4091
h2-ngd-x	3138	0.4534	0.8541	0.5808	0.8768	0.4008
SCD	34,705	0.4644	0.9817	0.1268	0.7053	0.9755
BigClam	4952	0.3778	0.4857	0.6807	0.9269	0.3121
Graclus	4633	0.4765	0.6915	0.6006	0.8852	0.4517
NISE	4903	0.4118	0.5735	0.7942	0.9518	0.3552

**Table 4:** *Amazon*: internal measures

Algorithm	Number of clusters	Coverage (%)	Algorithm time (s)	Total time (s)	Average Ncut
h2-n-a	4989	98.84	466.99	468.09	0.1657
h2-n-x	4988	98.80	452.05	453.13	0.1711
h2-ng-a	4990	98.73	537.82	538.91	0.1617
h2-ng-x	4988	98.66	514.71	515.81	0.1709
h2-ngd-a	4990	98.82	573.64	574.73	0.1491
h2-ngd-x	4990	98.79	560.86	561.96	0.1545
SCD	141,405	100.00	1.86	4.37	0.8418
BigClam	5000	97.31	N/A	169.51	0.3198
Graclus	5000	100.00	119.25	119.45	0.1450
NISE	5182	99.63	990.84	990.86	0.1118

**Table 5:** *Amazon*: external measures

Algorithm	Number of clusters	*F*1	Precision	Recall	Reverse precision	Reverse recall
h2-n-a	1069	0.7883	0.9747	0.8179	0.9057	0.7593
h2-n-x	1038	0.7717	0.9787	0.8109	0.9070	0.7311
h2-ng-a	1209	0.7422	0.9657	0.7247	0.8748	0.7622
h2-ng-x	1185	0.7268	0.9655	0.7152	0.8743	0.7372
h2-ngd-a	1181	0.7813	0.9698	0.7741	0.8867	0.7922
h2-ngd-x	1168	0.7725	0.9702	0.7681	0.8869	0.7792
SCD	3841	0.6202	0.9998	0.3166	0.8186	0.9948
BigClam	1447	0.8389	0.9718	0.7824	0.9574	0.8744
Graclus	991	0.8555	0.9356	0.9471	0.9892	0.7525
NISE	2612	0.6673	0.6666	0.9733	0.9807	0.5390

**Table 6:** *Youtube*: internal measures

Algorithm	Number of clusters	Coverage (%)	Algorithm time (s)	Total time (s)	Average Ncut
h2-n-a	3782	98.10	1182.39	1185.94	0.1681
h2-n-x	3780	98.01	1189.09	1192.66	0.1634
h2-ng-a	3798	98.00	1885.15	1888.71	0.1520
h2-ng-x	3851	98.14	1816.98	1820.45	0.1491
h2-ngd-a	3886	98.27	1613.13	1616.57	0.1395
h2-ngd-x	3874	98.22	1621.04	1624.50	0.1428
SCD	998,722	100.00	12.03	20.39	0.9882
BigClam	5000	41.51	N/A	2379.84	0.7398
Graclus	5000	100.00	2160.11	2168.36	0.4919
NISE	5162	99.96	2598.25	2598.66	0.4313

**Table 7:** *Youtube*: external measures

Algorithm	Number of clusters	*F*1	Precision	Recall	Reverse precision	Reverse recall
h2-n-a	189	0.2907	0.9639	0.5247	0.9810	0.0403
h2-n-x	193	0.2972	0.9645	0.5411	0.9790	0.0412
h2-ng-a	241	0.2935	0.8684	0.5969	0.9315	0.0516
h2-ng-x	259	0.3027	0.8932	0.5915	0.9467	0.0551
h2-ngd-a	227	0.3030	0.9299	0.5694	0.9594	0.0484
h2-ngd-x	238	0.2978	0.9394	0.5476	0.9633	0.0507
SCD	27,864	0.3652	0.9709	0.1330	0.4453	0.9841
BigClam	3850	0.2354	0.3755	0.5187	0.4743	0.2370
Graclus	3802	0.3827	0.5761	0.5348	0.6532	0.4148
NISE	3778	0.2720	0.4762	0.7180	0.9912	0.2580

**Table 8:** *DBLP15*: internal measures

Algorithm	Number of clusters	Coverage (%)	Algorithm time (s)	Total time (s)	Average Ncut
h2-n-a	4982	99.66	1648.73	1654.71	0.1702
h2-n-x	4982	99.67	1666.13	1672.01	0.1743
h2-ng-a	4984	99.62	3262.76	3268.63	0.1606
h2-ng-x	4984	99.64	3220.70	3226.57	0.1568
h2-ngd-a	4987	99.69	2558.80	2564.60	0.1457
h2-ngd-x	4987	99.70	2503.58	2509.38	0.1463
SCD	565,235	100.00	16.89	33.22	0.8357
BigClam	5000	65.07	N/A	1352.57	0.6761
Graclus	5000	100.00	1980.38	1987.97	0.2732
NISE	5101	86.77	945.15	945.90	0.3482

**Table 9:** *DBLP15*: external measures

Algorithm	Number of clusters	*F*1	Precision	Recall	Reverse precision	Reverse recall
h2-n-a	4982	0.3028	0.7282	0.7000	0.9830	0.0445
h2-n-x	4982	0.2994	0.7229	0.6986	0.9833	0.0442
h2-ng-a	4984	0.3025	0.7188	0.7164	0.9066	0.0440
h2-ng-x	4984	0.2992	0.6978	0.7275	0.9095	0.0439
h2-ngd-a	4987	0.3036	0.6963	0.7455	0.9640	0.0446
h2-ngd-x	4987	0.3016	0.6839	0.7512	0.9658	0.0446
SCD	565,235	0.3477	0.8684	0.1050	0.5803	0.8218
BigClam	5000	0.0784	0.2357	0.9875	0.6806	0.0192
Graclus	5000	0.0861	0.2411	0.9874	0.7576	0.0275
NISE	5101	0.0955	0.3606	0.8307	0.7066	0.0253

In the “internal measures” table, “coverage” measures the percentage of nodes which are assigned to at least one community; “algorithm time” and “total time” provide the runtime information. We list two measures of runtime since our algorithm (and also NISE) implemented in MATLAB directly uses a processed matrix in memory as its input. Other algorithms must first read the graph stored as an edge list or an adjacency list and convert the graph to the appropriate internal representation. Therefore, we use “algorithm time” to measure the algorithm runtime without the time needed for reading and converting the graph, which is reported by the algorithms themselves. The “total time” is the wall clock time for running the algorithm, including the time for reading and converting the graph, which is measured with an external timer. BigClam reports its algorithm time as the sum of time used in each core, and therefore, the results are not comparable. For completeness, we added the data-loading and data-preprocessing times, which are measured separately, to obtain a “total time” for the MATLAB algorithms (our algorithm and NISE).

The “number of clusters” in the “internal measures” table are different across different methods due to the following reasons. The SCD algorithm does not provide an interface for specifying the number of communities to detect, and instead detects the number of communities automatically. For other algorithms, we specify the number of communities to detect as 5000. The actual number of communities generated by HierSymNMF2 is usually less than 5000, as discussed in “Methods for comparison” section. Also, the number of communities generated by NISE are usually a little larger than 5000, which is also an expected behavior [3].

In the “external measures” table, the “reverse precision” and “reverse recall” refer to the scores computed as if the ground-truth communities are treated as detected communities and the detected communities are treated as the ground truth, respectively. Note that the number of clusters in “external measures” is less than the one in “internal measures” due to the removal of nodes that do not appear in the ground truth.

We have the following observations from the experimental results: (1) Our HierSymNMF2 algorithm has significant advantages over other methods in average normalized cut on most datasets except the *Amazon* dataset. On the *Amazon* dataset, HierSymNMF2 achieves much lower average normalized cut than SCD and BigClam, and the variant h2-ngd-a obtained comparable average normalized cut (0.1491) versus Graclus (0.1450), which is not as good as NISE (0.1118). (2) HierSymNMF2 runs slower than most other algorithms on *DBLP06* and *DBLP15*. On the *Youtube* dataset, HierSymNMF2 runs faster than BigClam, Graclus and NISE. On the *Amazon* dataset, HierSymNMF2 runs faster than NISE, but slower than other methods. (3) HierSymNMF2 achieves better *F*1 score than BigClam and NISE on all the datasets we used. Graclus has better *F*1 score than HierSymNMF2 on *DBLP06*, *Amazon*, and *Youtube* datasets but obtained an unusually low *F*1 score on the *DBLP15* dataset. SCD achieves higher *F*1 scores than HierSymNMF2. However, SCD often discovers a significantly larger number of (nonoverlapping) communities than expected and has very unbalanced precision and recall scores compared with other algorithms. The SCD algorithm finds the number of communities as it finds the communities and the number of communities cannot be given to SCD as an input. The SCD algorithm starts by assigning an initial partitioning of the graph heuristically. In short, in the initial partitioning, each node and all its neighbors form a community, and special care is taken to ensure that no node belongs to more than one community. As a result, this initial step often creates many more communities than the optimal number, although later refining procedures may reduce the number of communities. As can be seen from the experimental results, when compared with BigClam, Graclus, NISE, and our proposed algorithms that take the number of communities as an input, a much larger number of communities that the SCD generates does not necessarily translate to a better overall community detection result in terms of either normalized cut or *F*1 scores.

## Conclusions and discussion

Overall, HierSymNMF2 is an effective community detection method that optimizes the average normalized cut very well, although it is not the fastest method.

To address the quality issue of the ground-truth networks, we constructed a more complete and recent version of the DBLP dataset, where most nodes have at least one community membership and also the size of the data is significantly larger.

We partially answered the three questions we raised in “Problem definition” section. For Q1 and Q2, we used measurements that are commonly used in the current literature. One has to be careful when choosing community detection methods based on external measures such as *F*1 score because of the incompleteness of ground-truth communities and the difference between linkage-based communities (as detected) and functional communities (as in the ground truth). Therefore, it is important to use an internal measure such as the average normalized cut to evaluate an algorithm. In addition, we believe the current evaluation methods for large-scale community detection have certain limitations. These methods are mainly based on some quality scores, e.g., the *F*1 score. Such quality scores can be used to compare various algorithms. However, they do not provide much more information regarding the quality of results than the average performance. We assert that to better understand a community detection result, it is necessary to develop more comprehensive evaluation methods.

For Q3, we think that the large scale of the network and the large number of communities make the community detection results hard to interpret. One way to understand the community structure better would be to develop better methods for community visualization.

As a result of the growing popularity and utility of social media communications and other channels of communications between people and groups of people, there are vast amounts of data that contain latent community information. The amount of information is overwhelming and very demanding of our current technological capabilities, which may adversley impact the ability of stakeholders to make critical and timely decisions that are important in many domains such as natural disasters, local conflicts, healthcare, and law enforcement, to name a few. These domains typically involve groups of individuals with often hidden links. Thus, it is incumbent on the research community to develop fast and effective methods to first discover the communities formed by these links and then formulate useful summaries of the information provided by the algorithms and measures in order for decision makers to initiate appropriate actions as required.

## Appendix A: Relations between SymNMF, ratio association and normalized cut

We mentioned that the SymNMF formulation

$$\begin{aligned} \min _{H\ge 0} \Vert S-HH^\text{T}\Vert _F^2 \quad \quad \quad \quad \quad \quad (2 \;\mathrm{revisited}) \end{aligned}$$

is a relaxation of optimization problems related to graph partitioning. Specifically, let $S^{\mathcal {G}}$ be the adjacency matrix of graph $\mathcal {G}$. When $S=S^{\mathcal {G}}$, Eq. (2) is a relaxation of maximizing the ratio association. When $S=D^{-1/2}S^{\mathcal {G}}D^{-1/2}$, where $D = {\text {diag}}(d_1,\ldots ,d_n)$ and $d_i=\sum _{j=1}^n S^{\mathcal {G}}_{ij}$ is the degree of node *i*, Eq. (2) is a relaxation of minimizing the normalized cut.

We defined normalized cut of a graph partition $(A_1,\ldots ,A_k)$ as ${{\mathrm{ncut}}}(A_1,\ldots ,A_k)$ (Eq. (9)) using the concept of ${{\mathrm{within}}}(A_i)$ (Eq. (10)), and ${{\mathrm{out}}}(A_i)$ (Eq. (11)). With the same notations, the ratio association of a graph partition is defined as

15 $$\begin{aligned} {\text {rassoc}}(A_1,\ldots ,A_k) = \sum _{i=1}^k \frac{{{\mathrm{within}}}(A_i)}{|A_i|} \end{aligned}$$

where $|A_i|$ is the number of nodes in partition $A_i$.

In the following sections, we will use the convenient Iverson bracket [41]:

16 $$\begin{aligned}{}[P] = {\left\{ \begin{array}{ll} 1 & \quad \text {if } P \text { is true;} \\ 0 & \quad \text {otherwise.} \end{array}\right. } \end{aligned}$$

where *P* is any statement. Also, $\mathcal {G}=(V,E)$ is the graph under study, where $V=\{v_1,\ldots ,v_n\}$. The matrix $S=(w_{ij})$ is the adjacency matrix of graph $\mathcal {G}$, where $w_{ij}=w(v_i,v_j)$ is the weight of edge $(v_i,v_j)$. The tuple $(A_1,\ldots ,A_j)$ is a partition of graph $\mathcal {G}$.

### Relation between SymNMF and ratio association

We rewrite Eq. (15) as

17 $$\begin{aligned} {\text {rassoc}}(A_1,\ldots ,A_k)&= \sum _{i=1}^k \frac{1}{|A_i|}\sum _{u,v\in A_i} w(u,v) \end{aligned}$$

Let $H=(h_{ij})_{n\times k}$ where *n* is the number of nodes in the graph, *k* is the number of partitions, and

18 $$\begin{aligned} h_{ij} = \frac{1}{\sqrt{|A_j|}}[v_i\in A_j] \end{aligned}.$$

Then, we have

$$\begin{aligned} {\text {tr}}(H^\text{T}SH)&= \sum _{\begin{array}{c} 1\le i \le k\\ 1\le j,l\le n \end{array}}h_{ji}w_{jl}h_{li}\\&=\sum _{\begin{array}{c} 1\le i \le k\\ 1\le j,l\le n \end{array}} \frac{w_{jl}}{|A_i|}[v_j\in A_i][v_l\in A_i]\\&= \sum _{i=1}^k \frac{1}{|A_i|}\sum _{u,v\in A_i} w(u,v)\\&= {\text {rassoc}}(A_1,\ldots ,A_k) \end{aligned}$$

and

$$\begin{aligned} (H^\text{T}H)_{ij}&=\sum _{k}h_{ki}h_{kj}\\&=\sum _{k}\frac{1}{\sqrt{|A_i||A_j|}}[v_k\in A_i][v_k \in A_j]\\&=\frac{[i=j]}{|A_i|}\sum _{k}[v_k\in A_i]\\&=[i=j] \end{aligned}$$

which means $H^\text{T}H=I$. Therefore [42],

19 $$\begin{aligned} \max {\text {rassoc}}(A_1,\ldots ,A_k)&\Leftrightarrow \max {\text {tr}}(H^\text{T}SH)\nonumber \\&\Leftrightarrow \min \{{\text {tr}}(S^\text{T}S)-2{\text {tr}}(H^\text{T}SH)+ {\text {tr}}(I)\}\nonumber \\&\Leftrightarrow \min {\text {tr}} \left( (S-HH^\text{T})^\text{T}(S-HH^\text{T}) \right) \nonumber \\&\Leftrightarrow \min \Vert S-HH^\text{T}\Vert _F^2 \end{aligned}$$

If the restriction (18) is relaxed using $H\ge 0$ , i.e., nonnegative *H*, we will arrive at our SymNMF formulation.

### Relation between SymNMF and normalized cut

Let $D = {\text {diag}}(d_1,\ldots ,d_n)$ , where $d_i=\sum _{j=1}^n w_{ij}$ is the degree of node *i* and let $H=(h_{ij})_{n\times k}$, where

20 $$\begin{aligned} h_{ij}=\frac{\sqrt{d_i}}{\sqrt{{{\mathrm{within}}}(A_j)+{{\mathrm{out}}}(A_j)}} [v_i\in A_j] \end{aligned}.$$

Then, we have

$$\begin{aligned} {\text {tr}}(H^\text{T}D^{-1/2}SD^{-1/2}H)&= \sum _{\begin{array}{c} 1\le i \le k\\ 1\le j,l\le n \end{array}}\frac{h_{ji}w_{jl}h_{li}}{\sqrt{d_jd_l}}\\&=\sum _{\begin{array}{c} 1\le i \le k\\ 1\le j,l\le n \end{array}} \frac{w_{jl}}{{{\mathrm{within}}}(A_i)+{{\mathrm{out}}}(A_i)} [v_j\in A_i][v_l\in A_i]\\&= \sum _{i=1}^k \frac{{{\mathrm{within}}}(A_i)}{{{\mathrm{within}}}(A_i)+{{\mathrm{out}}}(A_i)}\\&= k-{{\mathrm{ncut}}}(A_1,\ldots ,A_k) \end{aligned}$$

and

$$\begin{aligned}&(H^\text{T}H)_{ij}\\&=\sum _{k}h_{ki}h_{kj}\\&=\sum _{k}\frac{d_k}{ \sqrt{{{\mathrm{within}}}(A_i)+{{\mathrm{out}}}(A_i)} \sqrt{{{\mathrm{within}}}(A_j)+{{\mathrm{out}}}(A_j)} }[v_k\in A_i\cap A_j]\\&=\frac{[i=j]}{{{\mathrm{within}}}(A_i)+{{\mathrm{out}}}(A_i)}\sum _{k}d_k[v_k\in A_i]\\&=[i=j] \end{aligned}$$

which means $H^\text{T}H=I$. Similar to (19), we have

21 $$\begin{aligned} \min {{\mathrm{ncut}}}(A_1,\ldots ,A_k)&\Leftrightarrow \min \{k-{\text {tr}}(H^\text{T}D^{-1/2}SD^{-1/2}H)\}\nonumber \\&\Leftrightarrow \max {\text {tr}}(H^\text{T}D^{-1/2}SD^{-1/2}H)\nonumber \\&\Leftrightarrow \min \Vert D^{-1/2}SD^{-1/2}-HH^\text{T}\Vert _F^2 \end{aligned}.$$

When the restriction (20) is relaxed to $H\ge 0$, our SymNMF formulation is obtained.

## Appendix B: Normalized cut increases when a community is split into two communities

In Fig. 1, the community $A_3$ was split into $B_1$ and $B_2$, and the associated increase of normalized cut is

$$\begin{aligned} \Delta _{\text{ncut}} &= \frac{{{\mathrm{out}}}(B_1)}{{{\mathrm{within}}}(B_1)+{{\mathrm{out}}}(B_1)}+ \frac{{{\mathrm{out}}}(B_2)}{{{\mathrm{within}}}(B_2)+{{\mathrm{out}}}(B_2)} \\ & \quad - \frac{{{\mathrm{out}}}(A_3)}{{{\mathrm{within}}}(A_3)+{{\mathrm{out}}}(A_3)} \end{aligned}.$$

Denote $i_1={{\mathrm{within}}}(B_1)$, $i_2={{\mathrm{within}}}(B_2)$, $i_3={{\mathrm{within}}}(A_3)$, $o_1={{\mathrm{out}}}(B_1)$, $o_2={{\mathrm{out}}}(B_2)$, $i_3={{\mathrm{out}}}(A_3),$ and $c={{\mathrm{cut}}}(B_1,B_2),$ and note that $o_3=o_1+o_2-2c$ and $i_3=i_1+i_2+2c$. Then, we have

$$\begin{aligned} \Delta _{\text{ncut}} =\frac{2c(i_1+o_1)(i_2+o_2)+o_2(i_1+o_1)^2+o_1(i_2+o_2)^2}{\left( i_1+o_1\right) \left( i_2+o_2\right) \left( i_1+i_2+o_1+o_2\right) } >0 \end{aligned}$$

Note that this proof does not say that more communities always correspond to larger normalized cut in general (i.e., when the communities are not obtained through recursive splitting).

## Abbreviations

NMF: nonnegative matrix factorization
SymNMF: symmetric nonnegative matrix factorization
LRA: low rank approximation
NLS: nonnegative least squares
ANLS: alternating nonnegative least squares
SpMM: sparse–dense matrix multiplication
SpMV: sparse matrix–vector multiplication
CSC: compressed sparse column
AVX: advanced vector extensions
SIMD: single instruction, multiple data
opt-act: optimal active set

## Footnotes

1
https://snap.stanford.edu/data/.

2 The more general form of SpMM is $Y \leftarrow Y + A \cdot X$. Our algorithm only requires the more simplistic form $Y \leftarrow A \cdot X$, and thus, for this case, we wrote a specific routine saving *n* operations for addition.

3
https://www.mathworks.com/.

4
http://eigen.tuxfamily.org.

5
http://arma.sourceforge.net/.

6
https://software.intel.com/en-us/intel-mkl.
